# Impact of a Scalable, Multi-Campus “Foodprint” Seminar on College Students’ Dietary Intake and Dietary Carbon Footprint

**DOI:** 10.3390/nu12092890

**Published:** 2020-09-22

**Authors:** Hannah Malan, Ghislaine Amsler Challamel, Dara Silverstein, Charlie Hoffs, Edward Spang, Sara A. Pace, Benji Lee Reade Malagueño, Christopher D. Gardner, May C. Wang, Wendelin Slusser, Jennifer A. Jay

**Affiliations:** 1 Department of Community Health Sciences, UCLA Fielding School of Public Health, Los Angeles, CA 90095, USA; maywang@ucla.edu (M.C.W.); wslusser@conet.ucla.edu (W.S.); 2 Menus of Change University Research Collaborative, Stanford, CA 94305, USA; gchallamel@stanford.edu; 3 Stanford Residential & Dining Enterprises, Stanford, CA 94305, USA; silverstein@stanford.edu; 4 Stanford Prevention Research Center, Stanford University, Palo Alto, CA 94305, USA; chuck99@stanford.edu (C.H.); benjilrm@stanford.edu (B.L.R.M.); cgardner@stanford.edu (C.D.G.); 5 Department of Food Science and Technology and the Center for Water-Energy Efficiency, University of California Davis, Davis, CA 95616, USA; esspang@ucdavis.edu (E.S.); sspace@ucdavis.edu (S.A.P.); 6 Semel Healthy Campus Initiative Center, University of California Los Angeles, Los Angeles, CA 90095, USA; 7 Department of Civil and Environmental Engineering, University of California Los Angeles, Los Angeles, CA 90095, USA; jennyjay@ucla.edu

**Keywords:** university students, dietary intake, eating behaviors, climate change, sustainable diets, seminar

## Abstract

Background: Dietary patterns affect both human health and environmental sustainability. Prior research found a ten-unit course on food systems and environmental sustainability shifted dietary intake and reduced dietary carbon footprint among college students. This research evaluated the impact of a similar, more scalable one-unit *Foodprint seminar* taught at multiple universities. Methods: We used a quasi-experimental pre-post nonequivalent comparison group design (*n* = 176). As part of the Menus of Change University Research Collaborative, research was conducted at three university campuses in California over four academic terms. All campuses used the same curriculum, which incorporates academic readings, group discussions, and skills-based exercises to evaluate the environmental footprint of different foods. The comparison group comprised students taking unrelated one-unit courses at the same universities. A questionnaire was administered at the beginning and end of each term. Results: Students who took the *Foodprint seminar* significantly improved their reported vegetable intake by 4.7 weekly servings relative to the comparison group. They also reported significantly decreasing intake of ruminant meat and sugar-sweetened beverages. As a result of dietary shifts, *Foodprint seminar* students were estimated to have significantly decreased their dietary carbon footprint by 14%. Conclusions: A scalable, one-unit *Foodprint seminar* may simultaneously promote environmental sustainability and human health.

## 1. Introduction

Dietary patterns affect both human health and environmental sustainability. The World Health Organization notes that a healthy diet is a sustainable diet and recommends a dietary pattern rich in plant-based foods with limited consumption of meat and meat products [1]. In addition, prominent scientists have recommended that high-income countries such as the United States increase consumption of plant-based foods and decrease consumption of red meat and sugar to ensure we can achieve the UN Sustainable Development Goals and maintain stability of the Earth system [2,3]. Climate change is currently a critical issue and threatens to exacerbate food insecurity and health disparities worldwide [4]. At the same time, the food system is a leading driver of climate change, contributing 25–30% of global greenhouse gas (GHG) emissions [4]. The livestock sector alone contributes 14.5% of GHG emissions, which is more than the entire transportation sector [5]. Ruminant livestock such as cows and sheep produce especially high amounts of GHG due to low energy conversion and enteric fermentation [6]. For example, one serving of beef has about 50 times the carbon footprint of a serving of beans [7].

Although there is high scientific agreement that we can simultaneously improve human health and environmental sustainability through dietary shifts, interventions targeting these shifts remain understudied [2,4,7,8,9]. Intervening in college and university settings is promising given these institutions’ (1) high levels of unilateral control, (2) broad reach, (3) pro-social missions, and (4) widespread commitment to promoting wellbeing [10,11,12,13]. Indeed, growing interest in university social responsibility (USR) may motivate higher education stakeholders to implement interventions targeting health and sustainability [14]. Koutali (2019) describes the potential of USR for developing agency among students and translating academic knowledge to broader society [15]. From a USR perspective, curricular interventions developed within universities are well positioned to scale across institutions and contexts [16].

Curricular interventions targeting dietary shifts are common in higher education; however, results have been mixed, and cognitive outcomes are typically stronger than behavioral outcomes [17]. Of note, two curricular interventions integrating the topics of food, environment, and society were found to be effective in shifting dietary intake among college students [18,19]. Jay et al. (2019) recently demonstrated the effectiveness of a two-quarter, ten-unit course about connections between food systems and environmental sustainability [18]. The study showed that students who took the course reported significantly reducing their ruminant meat consumption (i.e., beef and lamb), resulting in a lower dietary carbon footprint [18]. In addition, females who took the course reported significantly increasing their vegetable consumption [18]. Hekler et al. (2010) also found that college students who took a four-unit course on food and society significantly improved reported healthy eating behaviors [19].

Due to difficulties in scaling up large courses, we were interested in determining whether a one-unit course could yield impacts similar to Jay et al. (2019) and Hekler et al. (2010)—with potential for broader reach. One-unit courses may be more appealing to both instructors and students because they require less time investment. Thus, we developed and studied a one-unit seminar course taught at multiple universities through the Menus of Change University Research Collaborative (MCURC). MCURC was launched in 2014 as a working group of scholars, foodservice leaders, and administrators for colleges and universities (www.moccollaborative.org). In addition to promoting healthy, sustainable food in dining operations, the group studies various campus-based interventions. We hypothesized that a one-unit *Foodprint seminar* would result in: (1) healthy change in dietary intake, (2) a lower dietary carbon footprint, and (3) increased motivation for dietary behavior change.

## 2. Theoretical Background

Theory-based interventions tend to have stronger effects than those developed without theory [17]. The *Foodprint seminar*’s theory of change integrates concepts from the theory of planned behavior (TPB) and social cognitive theory [20,21]. Based on TPB, we hypothesized that participation in the course would shift participants’ dietary behavior by addressing beliefs and expectations about the outcomes of their food choices [20]. More specifically, we hypothesized that, by learning about the impacts of the food system on environmental sustainability, participants would increase value beliefs about the importance of environmental issues and increase intentions to engage in environmentally sustainable dietary behaviors (i.e., green eating intentions).

Recognizing that values and intentions may be insufficient for behavior change, the course also addressed the social cognitive theory principles of behavioral capability and self-efficacy [21]. We hypothesized that active learning activities would increase participants’ capability to evaluate the environmental impact of food and choose lower-impact foods. In turn, we hypothesized this would increase participants’ self-efficacy (i.e., confidence) to impact climate change through dietary shifts, resulting in lower ruminant meat consumption and dietary carbon footprint.

## 3. Materials and Methods

### 3.1. Study Design and Study Sites

This pilot study used a quasi-experimental pre-post nonequivalent comparison group design. Research was conducted at three university campuses, including the University of California Los Angeles (UCLA), Stanford University, and the University of California Davis (UCD), all members of MCURC. Students enrolled in the one-unit *Foodprint seminar* comprised the intervention group. With permission from instructors, comparison group students were recruited from eight other one-unit seminars unrelated to the *Foodprint seminar* content (e.g., civil engineering, psychology of successful aging, and Jane Austen). There was no random assignment, and students voluntarily enrolled in courses and participated in the study. All students were informed of the study prior to deciding whether or not to participate. The study took place over four academic terms: Fall 2018, Winter 2019, Spring 2019, and Fall 2019. Study procedures were approved by the lead University Institutional Review Board (UCLA—IRB#18–001468), and by the corresponding IRBs of the other participating schools.

### 3.2. Intervention Description: Foodprint Seminar

The intervention, *Foodprint seminar*, is a one-unit academic course on connections between food systems and environmental sustainability. Taught in a seminar-style format, course enrollment is capped at 20 to encourage discussion. At UCLA, the seminar was taught by a single professor; at Stanford by a team of one professor, the Dining Department Sustainable Program Manager, and two teaching assistants; and at UCD by a professor, a postdoctoral scholar, and four graduate students. All instructors used the same curriculum and communicated throughout the quarter to facilitate use of materials. Curriculum was delivered over one academic term with a one-hour seminar each week. An overview of the course topics is presented in Table 1. The *Foodprint seminar* incorporates academic readings, written reading reflections, group discussions, and skills-based active learning exercises. For example, students calculated the carbon, water, and land footprints at scales ranging from individual meals to the average diets of countries and presented their findings. The teaching format promoted critical thinking and group discussions through online platforms (e.g., Canvas) and during class sessions. All course materials, including class plans and detailed learning objectives, are available to view and download at healthy.ucla.edu/foodprint.

### 3.3. Data Collection

Data were collected at the beginning and end of each term during class (~10 min). Study staff invited students to participate and provided a link to a questionnaire administered online through SurveyMonkey (surveymonkey.com). Participation was optional, and no identifying information was collected. To maintain anonymity, students were asked to self-generate a code based on answers to four questions (e.g., “First letter of the month you were born”).

### 3.4. Key Outcome Measures

#### 3.4.1. Dietary Intake

Dietary intake was measured using a 39-item food frequency questionnaire (FFQ). The instrument was adapted from a validated FFQ designed to measure usual food group intake among university students (unpublished, see Supplementary Material S1). Students were asked to report how often they usually eat each item using a 10-point frequency scale, ranging from 0 = ”Never/rarely” to 9 = “Several times/day”. To aid with recall and reporting, examples were provided for some food item categories. For example, chicken, turkey, and duck were given as examples of “poultry”. Frequency responses were then standardized to servings per week and per day (see conversion factors in Supplementary Material S2).

Students were also asked to report how often they eat whole grains using three items with the phrasing: “When you eat [bread/rice/pasta or noodles], how often do you usually eat [whole-grain bread/whole-grain pasta or noodles/brown rice]?” Response options included: 4 = “Almost all the time”; 3 = “Some of the time”; 2 = “Not too often”; 1 = “Almost never”; 0 = “I do not eat [bread/rice/pasta or noodles]”. Composite scores were created by averaging responses >0. For example, if a student reported whole-grain bread = 0, whole-grain pasta or noodles = 2, and whole-grain rice = 4, the composite score was 3. Scores were then dichotomized, and scores ≥ 3 were identified as “Eats whole grains some/all the time.” Students who reported 0 for all three items (*n* = 2) were assigned a composite score of 0. To allow for stratified analysis of consumer subgroups, we created a dichotomous variable to identify those who reported consuming ≥ 1 serving of ruminant meat per week. We also created a green and healthy eating (GHE) score based on the USDA 2015–2020 Dietary Guidelines and Willett et al. (2019) planetary health diet [3,22]. This GHE score ranged from 0–4 with one point assigned for each of the following four criteria: >2 servings/day of fruit, ≥2.5 servings/day of vegetables for females/missing gender or ≥3 servings/day for males, ≤1 serving/week of red meat, and eats whole grains some/all the time.

#### 3.4.2. Dietary Carbon Footprint

Dietary carbon footprint calculations followed methodology reported in Jay et al. (2019) [18]. Prior to calculating carbon footprint, each student’s diet was normalized to 2000 kcal by summing daily servings across the 39 food items included in the FFQ. We assumed standard servings sizes and used gram and kcal values from the USDA Food Composition Database (fdc.nal.usda.gov). Each student’s reported intake was then adjusted using a unique multiplier to standardize calories to 2000 kcal/day. This approach was used to enable comparison of results to typical 2000 kcal/day diets discussed in the literature, while maintaining caloric distributions across reported food groups [23,24,25].

Normalized daily servings were then converted to grams of food per day and multiplied by carbon footprint conversion factors (g CO_2_-eq/g food). Published lifecycle assessment (LCA) data were used for carbon footprint conversion factors [18,23,26,27]. For multi-item food categories, we used average values. For example, “green vegetables” is the average of values available for broccoli, cabbage, collards/kale, lettuce, and spinach (see conversion factors in Supplementary Material S2). Dietary carbon footprint calculations are especially sensitive to conversion factors used for ruminant meat. In line with Jay et al. (2019), we used the beef value from Nijdam et al. (2012), which reflects typical production in the United States (40.2 g CO_2_-eq/g beef) [18,27]. We also calculated carbon footprints using the beef value from Heller and Keoleian (2014), which reflects a mean value for LCAs from developed countries (26.5 g CO_2_-eq/g beef) [23], and an average of values for beef and lamb from Nijdam et al. (2012) and Heller and Keoleian (2014) (29.8 CO2-eq/g ruminant meat) [23,27] (see calculations in Supplementary Material S3). Microsoft Excel Version 16.39 (Microsoft, Redmond, CA, USA) was used for all calculations.

#### 3.4.3. Psychosocial Outcomes

We assessed multiple psychosocial outcomes conceptualized as motivators for dietary behavior change: value beliefs, climate change self-efficacy, and green eating intentions. For value beliefs, we assessed (1) climate change, (2) environmental sustainability, (3) eating a healthful diet, (4) animal rights, and (5) social justice. Items were adapted from Hekler et al. (2010) and measured using a 6-point Likert importance scale, ranging from 0 = “Not at all important” to 5 = “The very most important.” The following phrasing was used: “Compared to other things in your life, please indicate the importance of [value]” [19].

Climate change self-efficacy is intended to capture an individual’s confidence in their ability to impact climate change through their own actions. According to social cognitive theory, self-efficacy determines whether individuals make use of the knowledge and skills they possess [28]. It is expected that those with higher climate change self-efficacy are more likely to take action on climate change [29]. We used three items adapted from Kellstedt et al. (2008) and asked students to rate their level of agreement with each statement using a 5-point Likert scale, ranging from 1 = “Strongly disagree” to 5 = “Strongly agree” [29]. Statements included (1) “I believe my actions have an influence on climate change”, (2) “My actions to reduce the effects of climate change in my community will encourage others to reduce the effects of climate change through their own actions”, and (3) “Human beings are responsible for climate change”. These three items were averaged to create a composite score, with higher scores indicating greater self-efficacy (Cronbach’s alpha = 0.68).

Green eating intentions were measured using five items adapted from Weller et al. (2014) and Monroe et al. (2015) [30,31]. According to the theory of planned behavior, intentions are the most important determinant of behavior [20]. In this research, intentions are conceptualized as indicators of motivational readiness to engage in green eating behaviors. More specifically, according to stages of change theory, intentions within a 6-month timeline indicate contemplation or awareness of the benefits of behavior change [32]. The following phrasing was used: “In the next 6 months, please indicate your intention to [behavior].” Behaviors included (1) “Choose local/seasonal foods”, (2) “Limit processed/fast foods”, (3) “Eat meatless meals at least once/week”, (4) “Choose organic foods when possible”, (5) “Take only what you plan on eating”. Responses were measured using a 5-point Likert scale, ranging from 1 = “Definitely not” to 5 = “Definitely yes”.

#### 3.4.4. Knowledge and Impact Self-Assessment

We added several items in Spring 2019 to provide insight into students’ change in knowledge and perceived course impact. Due to resource constraints, items were included only in the intervention group post survey. Items were developed based on Taylor-Powell and Renner’s (2009) guide to gathering post-intervention evaluation data [33]. For knowledge assessment, we used a valid retrospective design, where students were asked to rate their mastery of course competencies after and before the course. The list of competencies was developed by the course instructors. In additional closed-ended questions, students were asked to report how much of the course content they already knew and whether they intend to do anything differently as a result of the course. In open-ended questions, students were asked to report the most impactful thing they learned and what (if anything) they intend to do as a result of the course.

### 3.5. Statistical Analysis

Categorical variables were described as frequencies (%) and interval variables as means (standard deviations (SD)). All statistical analyses were conducted using Stata Statistical Software Version 15.1 (StataCorp LLC, College Station, TX, USA). Chi-square tests were used for bivariate analyses of all categorical variables. Non-parametric tests were used for interval variables because their distributions were skewed. To test for within-group changes from pre to post, we used Wilcoxon signed rank tests. To test for significant differences between the intervention and comparison groups at baseline, we used Wilcoxon Mann–Whitney tests. We also conducted difference-in-differences (DID) analyses to compare pre-post changes between groups. Wilcoxon Mann–Whitney tests were used for all DID analyses, except carbon footprint difference scores. Because carbon footprint difference scores were normally distributed, we used t-tests for DID analyses. We used a *p* < 0.05 significance level without adjustment for multiple testing. Because this is pilot study with a small sample size, we also identified results with *p* < 0.10 in tables to help guide future research questions.

## 4. Results

### 4.1. Study Participant Characteristics

Study participant characteristics are presented by study group in Table 2. There were no statistically significant differences between the comparison and intervention groups, except for academic quarter: A greater proportion of students in the comparison group participated in the study during the Fall quarter (67% vs. 52%, *p* = 0.04). The majority of participants attended the UCLA campus, participated in Fall quarter, are female, were in their first year of school, and are Asian or White race/ethnicity. Study participants include only those whose pre and post surveys were successfully matched. Loss to follow-up was substantial for both groups but greater for the comparison group. For the intervention group, loss ranged by quarter from 27% to 42% (mean = 34%), and for the comparison group, loss ranged from 51% to 62% (mean = 56%). Overall response rates were similar to those reported by Jay et al. (2019) [18].

### 4.2. Dietary Intake

Table 3 shows changes in reported dietary intake by study group, differences between groups at baseline, and differences in pre-post changes (i.e., difference-in-differences analysis). In general, the intervention group reported more environmentally sustainable dietary intake at baseline. Significant within group pre-post changes are noted in Table 3. Of the 16 dietary intake outcomes measured, the comparison group reported significant changes for two outcomes, and the intervention group reported significant changes for four outcomes. Students in the intervention group increased their vegetable intake by 2.1 servings/week, while students in the comparison group decreased their intake by 2.5 servings/week, resulting in a significant relative difference of 4.7 servings/week (*p* < 0.001). There was also a significant difference in grain intake change (*p* < 0.01): while the comparison group reported a decrease of about 5 weekly servings, the intervention group reported almost no change. GHE score also significantly improved among students in the intervention group relative to the comparison group (*p* < 0.01).

### 4.3. Ruminant Meat Intake and Dietary Carbon Footprint

Due to sensitivity of dietary carbon footprint to ruminant meat intake and significant group differences at baseline, we conducted overall and stratified analyses to determine intervention impacts on ruminant meat intake and dietary carbon footprint. For stratified analyses, students who reported consuming ruminant meat at least once/week at baseline were defined as weekly consumers, and those who reported consuming ruminant meat less than once/week at baseline were defined as infrequent consumers. As illustrated in Table 3 above, ruminant meat intake significantly differed between the groups at baseline. Approximately 52% (*n* = 46) of the intervention group and 71% (*n* = 62) of comparison group students were weekly ruminant meat consumers at baseline (*p* = 0.01).

Table 4 shows changes in ruminant meat intake stratified by weekly/infrequent ruminant meat intake at baseline. There were no significant baseline differences between groups for weekly (*p* = 0.76) or infrequent (*p* = 0.77) consumer subgroups. The decrease in ruminant meat intake among weekly consumers in the intervention group was significant relative to the comparison group (difference-in-differences = −0.9, *p* = 0.03). Changes among infrequent consumers were not statistically significant.

**Figure 1:**
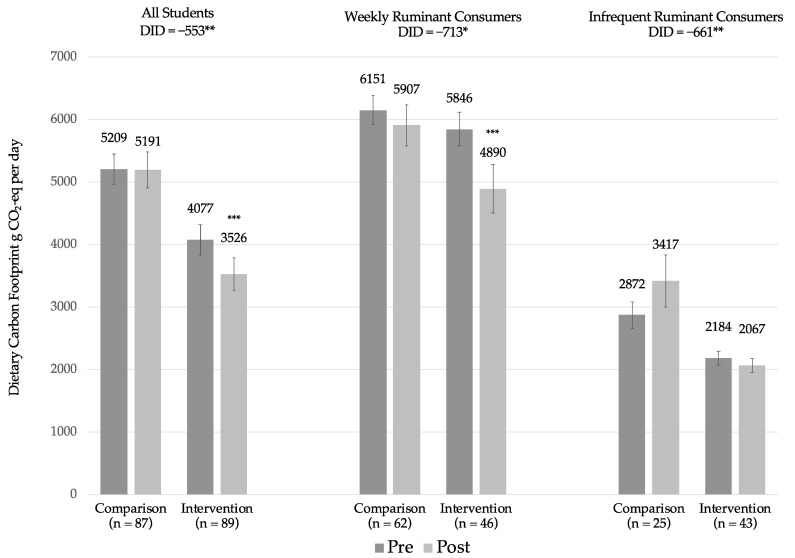
Pre and post daily dietary carbon footprint (g CO_2_-equivalent) by study group for all students, weekly ruminant consumers at baseline, and infrequent ruminant consumers at baseline. Note: Weekly consumers defined as those who reported consuming ruminant meat (beef/lamb) at least once/week at baseline. Infrequent consumers defined as those who reported consuming ruminant meat less than once/week at baseline. Within group pre-post changes tested using Wilcoxon signed rank tests. Difference-in-differences (DID) analysis of pre-post changes between groups tested using one-sided t-tests. * *p* < 0.10, ** *p* < 0.05, *** *p* < 0.01; error bars represent 95% confidence intervals.

Figure 1 shows mean daily dietary carbon footprint for a 2000-kcal normalized diet for all students, weekly ruminant meat consumers at baseline, and infrequent consumers at baseline. Among all students, baseline dietary carbon footprint was 5209 g CO_2_-eq for the comparison group and 4077 g CO_2_-eq for the intervention group; the baseline group difference of 1132 g CO_2_-eq was significant (*p* < 0.01). Decrease in dietary carbon footprint was significantly greater in the intervention group relative to the comparison group (difference-in-differences= −533, *p* = 0.04).

When stratifying by baseline ruminant meat intake, there were marked differences in dietary carbon footprint: In both groups, mean daily dietary carbon footprint of weekly ruminant meat consumers was about twice that of infrequent ruminant meat consumers. There were no significant baseline differences among weekly consumers (*p* = 0.64). Among infrequent ruminant meat consumers, dietary carbon footprint was significantly lower among students in the intervention group (*p* < 0.01). Among weekly consumers, the difference in change between groups approached significance (difference-in-differences = −713, *p* = 0.06). Among infrequent ruminant meat consumers, comparison group students increased, while intervention group students slightly decreased their dietary carbon footprint, resulting in a significant relative change (difference-in-differences = −661, *p* = 0.03).

### 4.4. Psychosocial Outcomes

Table 5 shows changes in psychosocial outcomes by study group, differences between groups at baseline, and differences in pre-post changes. In general, the intervention group reported greater motivational readiness for behavior change at baseline. Students in the comparison group reported no significant changes, while the intervention group reported significant changes in seven of the 11 outcomes measured. In difference-in-differences analyses, the intervention group reported significant improvements in three of the five green eating intentions relative to the comparison group; relative improvement in climate change self-efficacy score approached significance (*p* = 0.08).

### 4.5. Knowledge and Impact Self-Assessment

Knowledge and impact self-assessment data are provided for intervention group students from the Spring 2019 and Fall 2019 quarters (*n* = 60). When asked how much of the course content they already knew, the large majority of students (73%) reported “A little bit,” suggesting most information presented in the course was new to participants (Table 6). Table 7 shows students’ self-assessments of mastery of six core seminar topics at the start of the course (pre) and end of the course (post). All self-assessments were conducted at post; thus, assessments of pre-course knowledge were retrospective. Students reported significant improvements in all topic areas.

Students were also asked: “Do you intend to do anything differently as a result of this course?” Fifty-four out of 60 (90%) reported “Yes” (data not shown). Most of the open-ended comments describing these intentions revolved around reducing meat consumption. Other comments addressed reducing consumption of prepared/processed foods, shopping locally/seasonally, and reducing waste. Overall, open-ended comments corroborated results presented above showing significant increases in green eating intentions. Finally, students were asked to report the most impactful thing they learned in the course. Open-ended comments ranged from specific information about antibiotic use in livestock and the carbon footprint of ruminant products to more general awareness of the magnitude of the impact of the food system on environmental sustainability. All open-ended comments are provided in Supplementary Material S4.

## 5. Discussion

We found that a one-unit *Foodprint seminar* taught at multiple university campuses holds promise for shifting students’ dietary intake, reducing dietary carbon footprint, and increasing motivation for dietary behavior change. Regarding our first hypothesis (healthy change in dietary intake), we observed changes in line with recommended dietary shifts [3]. Specifically, students in the intervention group significantly decreased reported intake of ruminant meat and sugar-sweetened beverages (SSBs). Notably, students in the intervention group significantly improved reported vegetable intake by 4.7 weekly servings relative to the comparison group. Green and healthy eating (GHE) score also significantly improved among students in the intervention group relative to the comparison group. Unlike Jay et al. (2019), we found no significant gender differences in dietary changes [18].

Results also support the hypothesized decrease in dietary carbon footprint. Given the large contribution of ruminant meat intake to dietary carbon footprint, we examined changes overall and stratified by baseline ruminant meat intake. Overall, students in the intervention group significantly decreased reported ruminant meat intake by 0.7 servings per week (30%), and their daily dietary carbon footprint significantly decreased by an estimated 551 g CO_2_-eq (14%). Decrease in dietary carbon footprint was significant relative to the comparison group.

Among weekly ruminant meat consumers, students in the intervention group significantly decreased reported ruminant meat intake by 1.5 servings per week (35%). This decrease was significantly greater and more than double the 0.6 (14%) serving decrease reported among weekly consumers in the comparison group. Weekly consumers in the intervention group significantly decreased their estimated daily dietary carbon footprint by 957 g CO_2_-eq (16%). This change approached significance relative to the 244 g CO_2_-eq (4%) decrease observed among weekly consumers in the comparison group. We observed no significant changes among intervention group students who reported infrequent ruminant meat intake at baseline, which is likely attributable to a limited opportunity to shift to an even lower level (e.g., basement effect). However, infrequent consumers in the comparison group increased their dietary carbon footprint, resulting in a significant relative difference.

As discussed in Jay et al. (2019), dietary carbon footprint reductions can be understood in the context of broader carbon footprint reduction targets [18]. Under President Obama’s Climate Action Plan to meet the Paris Climate Accord, the United States aimed to reduce greenhouse gas emissions by 326 million metric tons per year (17% below 2005 levels by 2020) [34]. On a per capita basis, this amounts to approximately 2764 g CO_2_ per person per day. The significant 551 g CO_2_-eq reduction estimated among all students in the intervention group amounts to 20% of this target. Using the significant difference-in-differences value (−533 g CO_2_-eq), the improvement estimated among students in the intervention group relative to the comparison group amounts to 19% of the target.

In general, animal-based foods contribute more GHGs than plant-based foods; thus, shifting to a more plant-based diet reduces dietary carbon footprint [2]. For example, a study estimating the differences in dietary carbon footprint by dietary pattern in the United Kingdom (*n* > 55,000) found meat-eaters to be the highest, followed by fish-eaters, vegetarians, and vegans; the dietary carbon footprint of meat-eaters was approximately twice that of vegans [25]. Tilman and Clark (2014) also demonstrated through modeling that, compared to a projected 2050 global dietary pattern, shifting to Mediterranean, pescatarian, and vegetarian dietary patterns could reduce dietary carbon footprints by 30%, 45%, and 55%, respectively [9]. Future studies may consider the use of simulation modeling approaches to provide rigorous estimates of the potential climate change mitigation effects of more modest dietary shifts, such as those found in this study.

In support of hypothesis three, we found significant changes in psychosocial outcomes conceptualized as motivators for dietary behavior change. Intentions to choose local/seasonal foods, limit processed/fast foods, and eat meatless meals at least once/week significantly improved relative to the comparison group, indicating improvements in awareness of the benefits of these behaviors [32]. Finally, students in the intervention group reported significant improvements in mastery of all seminar topics measured, and 90% reported they intend to change their behavior as a result of the course.

The results of this pilot study suggest that a briefer, more scalable course implemented at multiple universities can yield impacts similar to those observed in Jay et al. (2019) and Hekler et al. (2010) [18,19]. In their study of a two-quarter, ten-unit course, Jay et al. (2019) observed similar, slightly smaller decreases in ruminant meat intake (28% in Jay et al. (2019) vs. 30% in this study) and dietary carbon footprint (7% in Jay et al. (2019) vs. 14% in this study) [18]. It should be noted we used an updated instrument for measuring dietary intake to improve accuracy; thus, differences in the magnitude of changes may reflect differences in measurement. Our study also provided insight into other dietary shifts, psychosocial outcomes, and learning/course impact outcomes described above. Healthy eating improvements, including increased reported vegetable intake and GHE score, were similar to those reported in Hekler et al. (2010) [19].

This research contributes to the literature on dietary interventions among college and university students and provides much needed evidence on strategies for promoting healthier, more environmentally sustainable diets. A recent systematic review of systematic reviews confirmed mixed effectiveness of university-based interventions delivered face-to-face in classrooms (*n* = 31 studies), with about half positively impacting dietary behavior [17]. At the same time, there is evidence that addressing dietary behavior from non-health-oriented perspectives may be particularly effective among this population [19,31,35,36,37]. For example, in their quasi-experiment, Hekler et al. (2010) found that students who took a course on food and society improved their healthy eating more than students in health-focused food courses (e.g., community health, obesity) [19]. In a qualitative study, university students identified social justice and environmental sustainability as motivators for developing and applying food literacy [35]. Surveys of young adults have found higher diet quality among those who value sustainable food production practices, and a brief online course was effective at increasing green eating behaviors among college students [36,37]. In short, educational interventions highlighting broader social and environmental impacts of food may build valuable awareness of food systems and contribute to individual-level behavior change.

It is worth discussing process motivation as a potential explanation for the effectiveness of non-health-oriented approaches to improve health-oriented behavior, such as dietary intake. Often referred to as the “stealth intervention” approach, Robinson’s (2010) theory of process motivation posits that health-beneficial interventions may be more effective by focusing on other rewarding aspects of a behavior—such as enjoyment or participation in a social movement—rather than health outcomes [38]. In the current study, students who took the *Foodprint seminar* were likely motivated to reduce their environmental footprint, which supported the reported adoption of health-beneficial dietary changes, such as increased vegetable intake and decreased red meat intake [39,40,41,42,43]. Intervention students also reported decreasing sugar-sweetened beverage intake and increasing intention to limit processed/fast food, both of which may protect against weight gain and chronic disease [44,45,46,47]. This process motivation logic is supported by reported increases in students’ knowledge of food systems, value of environmental sustainability, and climate change self-efficacy. Importantly, due to emphasis on intrinsic satisfaction, stealth interventions are expected to result in sustained interest and behavior change [38]. Future research should examine this hypothesis.

### Limitations and Strengths

There were several limitations to this research. First, participants were not randomly assigned to each group; thus, students predisposed to adopting healthier, more environmentally sustainable eating behaviors may have been more likely to enroll in the *Foodprint seminar*. Indeed, we found significant baseline differences between the comparison and intervention groups for dietary intake and motivational readiness outcomes, suggesting a threat of selection bias. Of note, baseline ruminant meat intake was significantly lower among the intervention group than the comparison group. In an attempt to provide more balanced comparisons, we presented stratified analyses by ruminant meat intake at baseline. Findings among weekly consumers suggest the effectiveness of the intervention to reduce ruminant meat intake among those with the highest potential for positive impact.

Second, all measures were self-reported and may be subject to social desirability and recall biases. To ensure anonymity, we used anonymous ID codes for matching pre and post surveys. To aid with dietary recall, we adapted a food frequency questionnaire designed for and validated for use with college students. When converting frequencies to servings, we assumed standard serving sizes, which may not capture true quantities consumed. However, errors in dietary measurement are likely evenly distributed across groups. Due to logistical and financial constraints, we were unable to conduct follow-up at additional time points. Additional time points of assessment would have been beneficial to determine the trajectory and sustainability of events.

Third, our sample size was relatively small (*n* < 90 in each group) due to the seminar-style class format and loss to follow-up. Enrollment shifts coupled with the lengthy questionnaire (~10 min) and voluntary participation likely contributed to loss of participants. In stratified analyses, subgroup sizes ranged from 25–62. Small sample sizes may have resulted in limited power to detect true difference-in-differences between groups. Furthermore, college is a natural period of transition in the life course, and new experiences are especially salient during the first months and year in school [14]. As such, it should be noted that a greater proportion of comparison students were in their first year of school and participated during Fall quarter. This may have contributed to changes observed in the comparison group and lack of clear intervention effects.

Fourth, generalizability is limited. Although multi-campus participation is a strength of the study, participants included students only from universities in California; thus, it is unknown whether the seminar would be effective in other locations and/or beyond the four-year university setting. Our sample was also largely female and Asian or White race/ethnicity. It would be valuable for future research to evaluate the seminar in other settings, with a larger and more diverse sample, with random assignment, and potentially in other formats as well (e.g., online course format).

This study also has several strengths. We recruited intervention and comparison group students from the same universities; thus, participants were exposed to similar contexts beyond the intervention, including the university food environment and campus culture. We also used a quasi-experimental, difference-in-differences approach to address threats to internal validity such as secular trends, interfering events, maturation, and seasonality [48]. Finally, this study is noteworthy in its collaborative nature and potential for intervention dissemination. The Menus of Change University Research Collaborative allowed this research to be conducted at multiple universities and facilitated communication, implementation, and continuous improvement of intervention materials for future use. The one-unit, seminar-style format of the course supports intervention feasibility due to relatively low investment requirements for both university instructors and participants alike.

## 6. Conclusions

Overall, findings suggest the one-unit *Foodprint seminar* simultaneously promotes environmental sustainability and human health by improving dietary intake and reducing dietary carbon footprint among college students. The significant dietary shifts reported in this study—including increased vegetable intake, decreased ruminant meat intake, and decreased SSB intake—are likely supported by students’ increased knowledge of connections between food systems and environmental sustainability, value of environmental sustainability, and climate change self-efficacy. This study supports prior research suggesting the effectiveness of “stealth interventions” (i.e., non-health-oriented) to improve health-related outcomes. The Menus of Change University Research Collaborative facilitated participation by multiple universities, allowing for broader intervention reach and collaboration among instructors. These results are promising given the demonstrated feasibility of providing the seminar at multiple campuses and potential for scalability.

## Figures and Tables

**Table 1:** Overview of *Foodprint seminar* course topics.

Week 1	Food and the planetary boundaries
Week 2	Climate change and the carbon footprint of food
Week 3	Food miles, packaging, and refrigeration
Week 4	Nutrient cycling
Week 5	Water—hidden water and bottled water
Week 6	Land use
Week 7	Biodiversity loss
Week 8	Chemical pollution—worker health, impacts on wildlife, and exposure to consumers
Week 9	Antibiotic resistance—humane treatment of animals
Week 10	Dietary shifts and sustainability

**Table 2:** Study participant characteristics by study group (*n* = 176).

Characteristics	Comparison (*n* = 87)		Intervention (*n* = 89)		*p*-Value
**Campus**	***n***	**%**	***n***	**%**	0.42
UCLA	59	67.8	55	61.8	
Stanford	20	23.0	20	22.5	
UC Davis	8	9.2	14	15.7	
**Academic Quarter**					0.04 *
Fall	58	66.7	46	51.7	
2018	16	27.6	16	34.8	
2019	42	72.4	30	65.2	
Winter 2019	14	16.1	13	14.6	
Spring 2019	15	17.2	30	33.7	
**Gender**					0.30
Female	55	63.2	63	70.8	
Male	32	36.8	25	28.1	
Refused	-	-	1	1.1	
**Year in School**					0.11
First	61	70.1	50	56.2	
Second	13	14.9	11	12.4	
Third	2	2.3	8	9.0	
Fourth/Fifth	11	12.7	20	22.5	
**Race/Ethnicity**					0.59
Asian	32	36.8	26	29.2	
White	30	34.5	33	37.1	
Hispanic/Latino	10	11.5	8	9.0	
Other/Multi	15	17.2	22	24.7	

* No significant differences between comparison and intervention groups using Chi-Square tests, except academic quarter (*p* = 0.04).

**Table 3:** Dietary intake outcomes by group and differences between groups (*n* = 176).

Dietary Intake Outcomes	Comparison (*n* = 87)			Intervention (*n* = 89)			Group Differences	
	Pre	Post	Diff ^a^	Pre	Post	Diff ^a^	Baseline ^b^	DID ^c^
**Food group servings/week, Mean (SD)**								
Fruit	10.4 (10.4)	8.4 (8.7)	−2.0 *	12.5 (9.5)	11.7 (8.1)	−0.9	2.2 **	1.1
Vegetables	19.9 (16.9)	17.3 (13.9)	−2.5 **	21.9 (14.7)	24.0 (14.6)	2.1 *	2.0	4.7 ***
Dairy	9.3 (9.1)	8.1 (8.9)	−1.2 *	6.7 (6.4)	5.6 (5.8)	−1.2 **	−2.6 *	0.0
Dairy alternatives	3.0 (5.3)	2.7 (5.0)	−0.3	4.9 (7.9)	4.6 (6.1)	−0.3	1.9 **	0.0
Animal-based protein	18.3 (13.0)	16.5 (13.2)	−1.8	15.0 (12.0)	12.7 (12.3)	−2.3 ***	−3.3 **	−0.5
Ruminant (beef/lamb)	3.1 (3.9)	2.8 (4.1)	−0.2 *	2.3 (3.4)	1.6 (3.0)	−0.7 ***	−0.8 **	−0.5
Pork	1.7 (2.0)	1.5 (2.7)	−0.1	1.4 (2.2)	1.1 (1.8)	−0.3	−0.3 **	−0.2
Poultry	7.0 (5.7)	6.1 (5.7)	−0.9 *	5.6 (5.9)	4.8 (5.7)	−0.8 *	−1.4 **	0.1
Fish/seafood	2.9 (4.0)	2.8 (4.0)	−0.1 *	2.0 (2.5)	1.8 (2.4)	−0.2 *	−0.9	−0.1
Eggs	3.6 (3.6)	3.2 (3.0)	−0.5	3.8 (3.7)	3.5 (3.6)	−0.3	0.2	0.2
Plant-based protein	12.0 (18.1)	9.3 (8.9)	−2.7	15.3 (12.7)	15.1 (13.1)	−0.2	3.3 ***	2.5
Grains	21.5 (17.9)	16.5 (13.5)	−5.0 ***	19.7 (11.6)	19.8 (11.5)	0.1	−1.8	5.1 ***
SSBs	1.7 (4.0)	1.7 (4.2)	0.0	1.2 (3.5)	0.8 (2.5)	−0.4 **	−0.5 **	−0.4
**Eat whole grains some/all the time (%)**	42.5	49.4	6.9	48.3	59.6	11.3	5.8	4.4
**Weekly ruminant intake (%)**	71.3	64.4	−6.9	51.7	40.5	−11.2	−19.6 **	−4.3
**GHE score ^d^, Mean (SD)**	1.41 (1.2)	1.38 (1.1)	−0.03	1.73 (1.3)	2.15 (1.2)	0.42	0.32	0.45 ***

Note: Numbers may not add up perfectly due to rounding. ^a^ Within group pre-post changes tested using Wilcoxon signed rank tests. ^b^ Baseline differences between intervention and comparison groups tested using Wilcoxon–Mann–Whitney and Chi-Square tests. ^c^ Difference-in-differences (DID) analysis of pre-post changes between groups tested using Wilcoxon–Mann–Whitney tests. ^d^ Green and healthy eating (GHE) score out of 4 points with 1 point for each: ≥2 servings/day fruit; ≥2.5 servings/day veg for female/missing and ≥3/day for male; ≤1 serving/week red meat; eats whole grains some/all the time; * *p* < 0.10, ** *p* < 0.05, *** *p* < 0.01.

**Table 4:** Ruminant meat outcomes by group and differences between groups, stratified by reported ruminant meat in take at baseline (*n* = 176).

Ruminant Meat Outcomes	Comparison (*n* = 87)			Intervention (*n* = 89)			Group Differences	
	Pre	Post	Diff ^a^	Pre	Post	Diff ^a^	Baseline ^b^	DID ^c^
**Servings/week, Mean (SD)**								
Weekly consumers	4.3 (4.0)	3.6 (4.3)	−0.6 **	4.3 (4.7)	2.8 (3.8)	−1.5 ***	0	−0.9 **
Infrequent consumers	0.2 (0.2)	0.9 (2.9)	0.7	0.1 (0.2)	0.2 (0.5)	0.1	−0.1	−0.6

Note: Numbers may not add up perfectly due to rounding. ^a^ Within group pre-post changes tested using Wilcoxon signed rank tests. ^b^ Baseline differences between intervention and comparison groups tested using Wilcoxon–Mann–Whitney and Chi-Square tests. ^c^ Difference-in-differences (DID) analysis of pre-post changes between groups tested using Wilcoxon–Mann–Whitney tests. Weekly consumers defined as those who reported consuming ruminant meat (beef/lamb) at least once/week at baseline, comparison group *n* = 62, intervention group *n* = 46. Infrequent consumers defined as those who reported consuming ruminant meat less than once/week at baseline, comparison group *n* = 25, intervention group *n* = 43. ** *p* < 0.05, *** *p* < 0.01.

**Table 5:** Mean (SD) psychosocial outcomes by group and differences between groups.

Psychosocial Outcomes	Comparison (*n* = 87)			Intervention (*n* = 89)			Group Differences	
	Pre	Post	Diff ^a^	Pre	Post	Diff ^a^	Baseline ^b^	DID ^c^
**Value Beliefs ^d^**								
Climate change	3.2 (1.3)	3.4 (1.2)	0.2	3.4 (1.2)	3.5 (1.1)	0.2 *	0.2	0.0
Environmental sustainability	2.8 (1.2)	2.9 (1.2)	0.1	3.3 (1.1)	3.5 (1.0)	0.2 **	0.5 **	0.1
Eating a healthful diet	3.1 (1.1)	3.3 (1.0)	0.2 *	3.5 (1.1)	3.7 (1.1)	0.2	0.5 **	0.0
Animal rights	2.4 (1.2)	2.3 (1.2)	0.0	2.5 (1.2)	2.5 (1.1)	0.0	0.1	0.0
Social justice	3.1 (1.2)	3.1 (1.3)	0.0	2.9 (1.2)	3.0 (1.1)	0.1	0.1	0.1
**Climate Change Self-Efficacy ^e^**								
Mean score	4.0 (0.7)	4.1 (0.7)	0.1	4.4 (0.5)	4.6 (0.4)	0.2 ***	0.4 ***	0.1 *
**Green Eating Intentions ^f^**								
Choose local/seasonal	3.6 (1.0)	3.6 (0.9)	0.0	3.8 (0.9)	4.1 (0.7)	0.3 ***	0.2	0.3 **
Limit processed/fast foods	4.1 (1.0)	4.1 (1.0)	0.0	4.3 (0.8)	4.6 (0.7)	0.2 ***	0.3 *	0.2 **
Eat meatless meals once/week	3.5 (1.4)	3.6 (1.4)	0.1	4.2 (1.2)	4.6 (0.8)	0.4 ***	0.7 ***	0.3 **
Choose organic when possible	3.5 (1.1)	3.6 (1.2)	0.1	3.8 (1.1)	4.1 (1.0)	0.2 **	0.3 *	0.1
Take only what plan on eating	4.6 (0.7)	4.6 (0.6)	0.0	4.6 (0.6)	4.8 (0.5)	0.2 **	0.1	0.2

Note: Numbers may not add up perfectly due to rounding. ^a^ Within group pre-post changes tested using Wilcoxon signed rank tests. ^b^ Baseline differences between intervention and comparison groups tested using Wilcoxon–Mann–Whitney tests. ^c^ Difference-in-difference (DID) analysis of pre-post changes between groups tested using Wilcoxon–Mann–Whitney tests. ^d^ Values measured on 6-point importance scale, ranging from 0 = “Not at all important” to 5 = “The very most important” with wording, “Compared to other things in your life, please indicate the importance of …”. ^e^ Climate change self-efficacy measured on 5-point scale, ranging from 1 = “Strongly disagree” to 5 = “Strongly agree”. ^f^ Green eating intentions measured on 5-point scale, ranging from 1 = “Definitely not” to 5 = “Definitely yes”; mean score calculated as mean of three efficacy beliefs measured; * *p* < 0.10, ** *p* < 0.05, *** *p* < 0.01.

**Table 6:** Intervention student responses to question: How much of the course content did you already know? (*n* = 60).

Already Knew	*n* (%)
None	1 (1.7)
A little bit	44 (73.3)
Quite a bit	14 (23.3)
All of it	1 (1.7)

Note: Data includes participants only from Spring 2019 and Fall 2019; thus, data are limited to *n* = 60.

**Table 7:** Mean (SD) self-assessment of mastery of seminar topics, intervention students (*n* = 60).

Seminar Topic	Pre	Post	Diff ***
Carbon footprint of food	2.2 (1.2)	3.5 (0.6)	1.30
Antibiotic resistance	1.8 (1.0)	3.2 (0.9)	1.42
Planetary boundaries	1.8 (1.0)	3.2 (0.8)	1.32
Biodiversity loss	1.9 (0.9)	3.0 (0.9)	1.08
Hid7den water	1.5 (0.9)	2.6 (0.9)	1.13
Nitrogen cycling	1.8 (1.0)	2.9 (0.9)	1.12

*** All pre-post differences significant at *p* < 0.001. Knowledge self-assessment conducted at post; students retrospectively assessed knowledge at pre. Self-assessments measured on a 4-point scale, ranging from 1 = “Not very well” to 4 = “Very well”. Note: Data includes participants only from Spring 2019 and Fall 2019; thus, data are limited to *n* = 60.

## Supplementary Materials

The following are available online at https://www.mdpi.com/2072-6643/12/9/2890/s1.

## References

[1] World Health Organization A Healthy Diet Sustainably Produced: Information Sheet. https://www.who.int/publications/i/item/WHO-NMH-NHD-18.12.

[2] Clark M., Springmann M., Hill J.D., Tilman D. (2019). Multiple health and environmental impacts of foods. Proc. Natl. Acad. Sci. USA.

[3] Willett W., Rockström J., Loken B., Springmann M., Lang T., Vermeulen S., Garnett T., Tilman D., Declerck F., Wood A. (2019). Food in the Anthropocene: The EAT–Lancet Commission on healthy diets from sustainable food systems. Lancet.

[4] Intergovernmental Panel on Climate Change (2019). Special Report on Climate Change and Land. https://www.ipcc.ch/srccl/.

[5] Food and Agrigulture Organization of the United Nations Livestock and the Environment. http://www.fao.org/livestock-environment/en/.

[6] Springmann M., Clark M., Mason-D’Croz D., Wiebe K.D., Bodirsky B.L., Lassaletta L., De Vries W., Vermeulen S.J., Herrero M., Carlson K.M. (2018). Options for keeping the food system within environmental limits. Nature.

[7] Harwatt H., Sabaté J., Eshel G., Soret S., Ripple W. (2017). Substituting beans for beef as a contribution toward US climate change targets. Clim. Chang..

[8] Springmann M., Wiebe K., Mason-D’Croz D., Sulser T.B., Rayner M., Scarborough P. (2018). Health and nutritional aspects of sustainable diet strategies and their association with environmental impacts: A global modelling analysis with country-level detail. Lancet Planet. Health.

[9] Tilman D., Clark M. (2014). Global diets link environmental sustainability and human health. Nature.

[10] Deliens T., Van Crombruggen R., Verbruggen S., De Bourdeaudhuij I., Deforche B., Clarys P. (2016). Dietary interventions among university students: A systematic review. Appetite.

[11] Kelly N.R., Mazzeo S.E., Bean M.K. (2013). Systematic Review of Dietary Interventions With College Students: Directions for Future Research and Practice. J. Nutr. Educ. Behav..

[12] National Center for Education Statistics Back to School Statistics. https://nces.ed.gov/fastfacts/display.asp?id=372.

[13] Nelson M.C., Story M., Larson N.I., Neumark-Sztainer D., Lytle L.A. (2008). Emerging Adulthood and College-aged Youth: An Overlooked Age for Weight-related Behavior Change. Obesity.

[14] Meseguer-Sánchez V., Abad-Segura E., Belmonte-Ureña L.J., Moreno V.M. (2020). Examining the Research Evolution on the Socio-Economic and Environmental Dimensions on University Social Responsibility. Int. J. Environ. Res. Public Health.

[15] Kouatli I. (2019). The contemporary definition of university social responsibility with quantifiable sustainability. Soc. Responsib. J..

[16] De Velazco J.J.H.G., Ravina-Ripoll R., Hernandez A.C.C. (2020). Relevance and social responsibility of sustainable university organizations: Analysis from the perspective of endogenous capacities. Entrep. Sustain. Issues.

[17] Belogianni K., Baldwin C. (2019). Types of Interventions Targeting Dietary, Physical Activity, and Weight-Related Outcomes among University Students: A Systematic Review of Systematic Reviews. Adv. Nutr..

[18] Jay J.A., D’Auria R., Nordby J.C., Rice D.A., Cleveland D.A., Friscia A., Kissinger S., Levis M., Malan H., Rajagopal D. (2019). Reduction of the carbon footprint of college freshman diets after a food-based environmental science course. Clim. Chang..

[19] Hekler E.B., Gardner C.D., Robinson T.N. (2010). Effects of a College Course About Food and Society on Students’ Eating Behaviors. Am. J. Prev. Med..

[20] Ajzen I. (1991). The theory of planned behavior. Organ. Behav. Hum. Decis. Process..

[21] Bandura A. (1986). Social Foundations of Thought and Action: A Social Cognitive Theory.

[22] U.S. Department of Health and Human Services, U.S. Department of Agriculture (2015). 2015–2020 Dietary Guidelines for Americans.

[23] Heller M., Keoleian G.A. (2014). Greenhouse Gas Emission Estimates of U.S. Dietary Choices and Food Loss. J. Ind. Ecol..

[24] Meier T., Christen O. (2012). Environmental Impacts of Dietary Recommendations and Dietary Styles: Germany As an Example. Environ. Sci. Technol..

[25] Scarborough P., Appleby P.N., Mizdrak A., Briggs A.D.M., Travis R.C., Bradbury K.E., Key T.J. (2014). Dietary greenhouse gas emissions of meat-eaters, fish-eaters, vegetarians and vegans in the UK. Clim. Chang..

[26] Drewnowski A., Rehm C., Martin A., Verger E., Voinnesson M., Imbert P. (2014). Energy and nutrient density of foods in relation to their carbon footprint. Am. J. Clin. Nutr..

[27] Nijdam D., Rood T., Westhoek H. (2012). The price of protein: Review of land use and carbon footprints from life cycle assessments of animal food products and their substitutes. Food Policy.

[28] Bandura A. (1998). Health promotion from the perspective of social cognitive theory. Psychol. Health.

[29] Kellstedt P.M., Zahran S., Vedlitz A. (2008). Personal Efficacy, the Information Environment, and Attitudes Toward Global Warming and Climate Change in the United States. Risk Anal..

[30] Weller K.E., Greene G., Redding C.A., Paiva A.L., Lofgren I., Nash J.T., Kobayashi H. (2014). Development and Validation of Green Eating Behaviors, Stage of Change, Decisional Balance, and Self-Efficacy Scales in College Students. J. Nutr. Educ. Behav..

[31] Monroe J.T., Lofgren I.E., Sartini B.L., Greene G.W. (2015). The Green Eating Project: Web-based intervention to promote environmentally conscious eating behaviours in US university students. Public Health Nutr..

[32] Ni Mhurchu C., Margetts B.M., Speller V.M. (2009). Applying the Stages-of-Change Model to Dietary Change. Nutr. Rev..

[33] Taylor-Powell E., Renner M. (2009). Collecting Evaluation Data: End-of-Session Questionnaires.

[34] The White House (2015). President Obama’s Climate Action Plan 2nd Anniversary Progress Report. https://obamawhitehouse.archives.gov/sites/default/files/docs/cap_progress_report_final_w_cover.pdf.

[35] Malan H., Watson T.D., Slusser W., Glik D., Rowat A.C., Prelip M. (2020). Challenges, Opportunities, and Motivators for Developing and Applying Food Literacy in a University Setting: A Qualitative Study. J. Acad. Nutr. Diet..

[36] Robinson-O’Brien R., Larson N., Neumark-Sztainer D., Hannan P., Story M. (2009). Characteristics and Dietary Patterns of Adolescents Who Value Eating Locally Grown, Organic, Nongenetically Engineered, and Nonprocessed Food. J. Nutr. Educ. Behav..

[37] Pelletier J.E., Laska M.N., Neumark-Sztainer D., Story M. (2013). Positive attitudes toward organic, local, and sustainable foods are associated with higher dietary quality among young adults. J. Acad. Nutr. Diet..

[38] Robinson T.N. (2010). Stealth interventions for obesity prevention and control: Motivating behavior change. Obesity Prevention.

[39] Bouvard V., Loomis D., Guyton K.Z., Grosse Y., El Ghissassi F., Benbrahim-Tallaa L., Guha N., Mattock H., Straif K. (2015). Carcinogenicity of consumption of red and processed meat. Lancet Oncol..

[40] Heidemann C., Schulze M.B., Franco O.H., Van Dam R.M., Mantzoros C.S., Hu F.B. (2008). Dietary patterns and risk of mortality from cardiovascular disease, cancer, and all causes in a prospective cohort of women. Circulation.

[41] Larsson S.C., Orsini N. (2013). Red Meat and Processed Meat Consumption and All-Cause Mortality: A Meta-Analysis. Am. J. Epidemiol..

[42] Sun Q., Pan A., Bernstein A.M., Schulze M.B., Manson J.E., Stampfer M.J., Willett W.C., Hu F.B. (2012). Red Meat Consumption and Mortality: Results from 2 prospective cohort studies. Arch. Intern. Med..

[43] Wang X., Ouyang Y., Liu J., Zhu M., Zhao G., Bao W., Hu F.B. (2014). Fruit and vegetable consumption and mortality from all causes, cardiovascular disease, and cancer: Systematic review and dose-response meta-analysis of prospective cohort studies. BMJ.

[44] Allman-Farinelli M., Partridge S., Roy R. (2016). Weight-Related Dietary Behaviors in Young Adults. Curr. Obes. Rep..

[45] Malik V.S., Popkin B., Bray G.A., Després J.-P., Hu F.B. (2010). Sugar-sweetened beverages, obesity, type 2 diabetes mellitus, and cardiovascular disease risk. Circulation.

[46] Poti J.M., Mendez M.A., Popkin B., Popkin B.M. (2015). Is the degree of food processing and convenience linked with the nutritional quality of foods purchased by US households?. Am. J. Clin. Nutr..

[47] Wang Y.C., Ludwig D.S., Sonneville K., Gortmaker S.L. (2009). Impact of Change in Sweetened Caloric Beverage Consumption on Energy Intake Among Children and Adolescents. Arch. Pediatr. Adolesc. Med..

[48] Rossi P.H., Lipsey M.W., Freeman H.E. (2004). Evaluation: A Systematic Approach.

