# Supplementary material for: Impact of a Scalable, Multi-Campus “Foodprint” Seminar on College Students’ Dietary Intake and Dietary Carbon Footprint
Source: Nutrients. 2020 Sep 22;12(9):2890. doi: 10.3390/nu12092890 (PMC7551495; doi:10.3390/nu12092890)
Supplement: Supplementary file 1 [file nutrients-12-02890-s001.zip › Foodprint S1.docx]

**Supplement 1. Food frequency questionnaire (FFQ) validation and adaptation**

The original food frequency questionnaire (FFQ) was developed by Wang, Cowgill, and Jung in 2016 based on the USDA 2015-2020 Dietary Guidelines and in collaboration with a university dietitian. The FFQ contained 34 items and was designed for university students with specific goals of (1) assessing usual food group intake in approximately ten minutes; (2) distinguishing plant protein intake from intake of meat/poultry/eggs. Students were asked to report how often they usually eat each item using a 10-point frequency scale: 0=“Never/rarely”; 1=“Once/month”; 2=“Twice/month”; 3=“Once/week”; 4=“Twice/week”; 5=“Few times/week”; 6=“5-6 times/week”; 7=“Once/day”; 8 =“Twice/day”; 9=”Several times/day.” To assess whole grain intake, three items were included the with phrasing: “When you eat [bread/rice/pasta or noodles], how often do you usually eat [whole-grain bread/whole-grain pasta or noodles/brown rice]?” Response options included: 4=“Almost all the time”; 3=“Some of the time”; 2=“Not too often”; 1=“Almost never”; 0=“I do not eat [bread/rice/pasta or noodles].”

**Table S1a.** Spearman correlations between food diary and FFQ food group estimates (n=41).

|  | **Coefficient** | **p-value** |
| --- | --- | --- |
| **Fruit** | .649 | <.001 |
| **Vegetables** | .418 | .007 |
| **Dairy** |  |  |
| Dairy | .421 | .006 |
| **Protein Foods** |  |  |
| Meat/Poultry/Eggs | .335 | .032 |
| Plant-Based Protein | .377 | .015 |
| Seafood | .291 | .065 |
| **Grains** |  |  |
| Grains | .224 | .160 |
| Whole Grains | .317 | .044 |
|  | | |

Note: Fruit and vegetable food groups do not include juice; vegetable group does not include French fries.

Following pretesting with seven students, the FFQ was revised to improve formatting and reduce respondent burden. To evaluate relative validity of the FFQ, food group and subgroup estimates were compared to matched three-day food diaries for a convenience subsample of 41 students. Food diary data were entered into the USDA SuperTracker tool (choosemyplate.gov/tools-supertracker) by trained public health graduate students to create food group totals. Spearman correlation coefficients ranged from 0.22 (p=.160) for grains to 0.65 (p<.001) for fruit, and all categories except seafood and grains exceeded 0.30. Correlation coefficients for all food groups assessed in the validation study are presented in Table S1a.

The adapted FFQ was slightly expanded to more accurately capture dietary carbon footprint. For example, while the original FFQ listed only one item for red meat, we listed two separate items—ruminant meats (e.g. beef, veal, goat, mutton, lamb) and pork—given the sensitivity of dietary carbon footprint to ruminant meat intake. We also separated cheese from yogurt and added additional items for meat and dairy alternatives, beverages, and grains. A complete list of food groups and items is included in Table S1b.

**Table S1b.** Food groups and items included in the original FFQ and adapted FFQ.

| Food group | Original FFQ item  (34 items) | Adapted FFQ item  (39 items) | Item # |
| --- | --- | --- | --- |
| **Fruit** | Fresh fruit | Temperate fruits (apple, blueberry, grapes, peaches, raspberry, strawberry) | 1 |
|  |  | Tropical fruits (avocado, lemon, kiwi, mango, orange, passionfruit) | 2 |
| **(Not included in fruit food group)** | 100% fruit juices | 100% fruit juices | 3 |
| **Vegetables** | Vegetables- green and leafy (e.g. broccoli, cabbage, bok choy) | Vegetables - green and leafy (e.g. broccoli, cabbage, salad greens, bok choy) | 4 |
|  | Other vegetables (e.g. carrots, jicama, radish, squash, potatoes NOT including French Fries) | Other vegetables (e.g. tomatoes, carrots, peppers, jicama, squash, potatoes NOT including French Fries) | 5 |
| **(Not included in vegetables food group)** | French Fries | French Fries | 6 |
|  | Vegetable juices | Vegetable juices | 7 |
| **Meat/Poultry/Eggs** | Poultry (e.g. chicken, turkey, duck) | Poultry (e.g. chicken, turkey, duck) | 8 |
|  | Red meat (e.g. beef, pork, lamb) | Ruminant meats  (e.g. beef, veal, goat, mutton, lamb) | 9 |
|  | Eggs | Pork or bacon | 10 |
|  |  | Eggs | 11 |
| **Seafood** | Fish (e.g. salmon, tuna) | Fish (e.g. salmon, tilapia, tuna) | 12 |
|  | Other seafood and shell-fish (e.g. shrimp, clams, mussels) | Other seafood and shell-fish (e.g. shrimp, clams, mussels) | 13 |
| **Plant-Based Protein** | Nut butter (e.g. peanut butter, almond butter) | Nut butter (e.g. peanut butter, almond butter) | 14 |
|  | Lentils | Lentils | 15 |
|  | Tofu, Seitan, and Tempeh | Tofu, Seitan, and Tempeh | 16 |
|  | Beans (e.g. kidney, garbanzo) | Beans (e.g. black, pinto, kidney, garbanzo) | 17 |
|  | Nuts and/or Seeds (e.g. almonds, walnuts, sunflower seeds, flax seed) | Nuts and/or Seeds (e.g. almonds, walnuts, sunflower seeds, flax seed) | 18 |
|  |  | Meat alternative products (e.g. veggie hot dogs,  veggie sausage, chickenless tenders) | 19 |
| **Dairy** | Milk | Dairy milk | 20 |
|  | Yogurt or cheese | Dairy yogurt | 21 |
|  | Yogurt drinks or fermented milk drinks | Cheese | 22 |
|  |  | Soy milk | 23 |
|  |  | Almond milk | 24 |
|  |  | Other vegan milk or cheese | 25 |
| **Grains** | Bread | Bread | 26 |
|  | Rice | Rice | 27 |
|  | Pasta or noodles | Pasta or noodles | 28 |
|  | Breakfast cereals (hot or cold) | Breakfast cereals (hot or cold) | 29 |
|  | Other grains (e.g. quinoa, farro, buckwheat, bulgar wheat, millet) | Other grains (e.g. quinoa, farro, buckwheat, bulgar wheat, millet) | 30 |
|  |  | Bagels | 31 |
|  |  | Tortillas | 32 |
| **Other (not included in validation study)** | Soda and other sugar-sweetened beverages | Soda and other sugar-sweetened beverages | 33 |
|  | Diet soda and other beverages containing artificial sweeteners | Diet soda and other beverages containing artificial sweeteners | 34 |
|  |  | Coffee | 35 |
|  |  | Tea | 36 |
|  |  | Bottled water (as opposed to tap) | 37 |
|  | Sweets and desserts (e.g. candy, cake, pie, ice cream) | Sweets and desserts (e.g. candy, cake, pie, ice cream) | 38 |
|  | Salty snacks (e.g. potato chips, tortilla chips, pretzels) | Salty snacks (e.g. potato chips, tortilla chips, pretzels) | 39 |

**Acknowledgements:** We would like to thank the following UCLA public health faculty and graduate students who contributed to the development and validation of the original food frequency questionnaire: Professor May Wang, Adjunct Assistant Professor Burton Cowgill, Associate Vice Provost Wendelin Slusser, Shelley Jung, Miranda Westfall, Courtnie Ly, Maliya Lor, Jasmine Mercado, Esther Gao, Sakura Takahashi, and Jocelyn Harrison.
