# Supplementary material for: Impact of a Scalable, Multi-Campus “Foodprint” Seminar on College Students’ Dietary Intake and Dietary Carbon Footprint
Source: Nutrients. 2020 Sep 22;12(9):2890. doi: 10.3390/nu12092890 (PMC7551495; doi:10.3390/nu12092890)
Supplement: Supplementary file 1 [file nutrients-12-02890-s001.zip › Foodprint S3.docx]

**Supplement 3. Dietary carbon footprint calculations using alternate values for ruminant meat**

**Table S3a**. Mean (SD) daily dietary carbon footprint (g CO_2_-equivalent) by group and differences between groups for all students and stratified by reported ruminant meat intake at baseline using 40.2 value for beef (reported in manuscript).

|  | **Comparison (n=87)** | | | | **Intervention (n=89)** | | | | **Group Differences** | | |
| --- | --- | --- | --- | --- | --- | --- | --- | --- | --- | --- | --- |
|  | **Pre** | **Post** | **Diff^a^** | **Pre** | | **Post** | **Diff^a^** | **Baseline^b^** | | **DID^c^** |  |
| **All students** | 5209 (2231) | 5191 (2681) | -17 | 4077 (2319) | | 3526 (2412) | -551*** | -1132*** | | -533** |  |
|  |  |  |  |  | |  |  |  | |  |  |
| **Baseline ruminant intake** | |  |  |  | |  |  |  | |  |  |
| Weekly consumers | 6151 (1850) | 5907 (2564) | -244 | 5846 (1839) | | 4890 (2633) | -957*** | -304 | | -713* |  |
| Infrequent consumers | 2872 (1070) | 3417 (2110) | 545 | 2184 (734) | | 2067 (744) | -116 | -689*** | | -661** |  |
|  | | | | | | | | | | | |

Note: Numbers may not add up perfectly due to rounding. ^a^Within group pre-post changes tested using Wilcoxon signed rank tests. ^b^Baseline differences between intervention and comparison groups tested using Wilcoxon-Mann-Whitney and Chi-Square tests. ^c^Difference-in-differences (DID) analysis of pre-post changes between groups tested using one-sided t-tests. Weekly consumers defined as those who reported consuming ruminant meat (beef/lamb) at least once/week at baseline, comparison group n=62, intervention group n=46; Infrequent consumers defined as those who reported consuming ruminant meat less than once/week at baseline, comparison group n=25, intervention group n=43. *p<.10, **p<.05, ***p<.01.

**Table S3b.** Mean (SD) daily dietary carbon footprint (g CO_2_-equivalent) by group and differences between groups for all students and stratified by reported ruminant meat intake at baseline using 26.5 value for beef.

|  | **Comparison (n=87)** | | | | **Intervention (n=89)** | | | | **Group Differences** | | |
| --- | --- | --- | --- | --- | --- | --- | --- | --- | --- | --- | --- |
|  | **Pre** | **Post** | **Diff** | **Pre** | | **Post** | **Diff** | **Baseline** | | **DID** |  |
| **All students** | 4482 (1660) | 4436 (1962) | -45 | 3557 (1762) | | 3127 (1796) | -429*** | -925*** | | -384** |  |
|  |  |  |  |  | |  |  |  | |  |  |
| **Baseline ruminant intake** | |  |  |  | |  |  |  | |  |  |
| Weekly consumers | 5158 (1364) | 4951 (1870) | -206 | 4881 (1382) | | 4173 (1891) | -707*** | -277 | | -501* |  |
| Infrequent consumers | 2806 (1018) | 3162 (1588) | 355 | 2142 (703) | | 2009 (669) | -133 | -664*** | | 488** |  |
|  | | | | | | | | | | | |

Note: Numbers may not add up perfectly due to rounding. ^a^Within group pre-post changes tested using Wilcoxon signed rank tests. ^b^Baseline differences between intervention and comparison groups tested using Wilcoxon-Mann-Whitney and Chi-Square tests. ^c^Difference-in-differences (DID) analysis of pre-post changes between groups tested using one-sided t-tests. Weekly consumers defined as those who reported consuming ruminant meat (beef/lamb) at least once/week at baseline, comparison group n=62, intervention group n=46; Infrequent consumers defined as those who reported consuming ruminant meat less than once/week at baseline, comparison group n=25, intervention group n=43. *p<.10, **p<.05, ***p<.01.

**Table S3c.** Mean (SD) daily dietary carbon footprint (g CO_2_-equivalent) by group and differences between groups for all students and stratified by reported ruminant meat intake at baseline using 29.8 value for beef.

|  | **Comparison (n=87)** | | | | **Intervention (n=89)** | | | | **Group Differences** | | |
| --- | --- | --- | --- | --- | --- | --- | --- | --- | --- | --- | --- |
|  | **Pre** | **Post** | **Diff** | **Pre** | | **Post** | **Diff** | **Baseline** | | **DID** |  |
| **All students** | 4657 (1793) | 4619 (2131) | -39 | 3682 (1894) | | 3224 (1942) | -459*** | -975*** | | -420** |  |
|  |  |  |  |  | |  |  |  | |  |  |
| **Baseline ruminant intake** | |  |  |  | |  |  |  | |  |  |
| Weekly consumers | 5397 (1475) | 5181 (2033) | -216 | 5113 (1487) | | 4345 (2066) | -768*** | -284 | | -552* |  |
| Infrequent consumers | 2822 (1029) | 3223 (1708) | 401 | 2152 (710) | | 2023 (686) | -129 | -670*** | | -529** |  |
|  | | | | | | | | | | | |

Note: Numbers may not add up perfectly due to rounding. ^a^Within group pre-post changes tested using Wilcoxon signed rank tests. ^b^Baseline differences between intervention and comparison groups tested using Wilcoxon-Mann-Whitney and Chi-Square tests. ^c^Difference-in-differences (DID) analysis of pre-post changes between groups tested using one-sided t-tests. Weekly consumers defined as those who reported consuming ruminant meat (beef/lamb) at least once/week at baseline, comparison group n=62, intervention group n=46; Infrequent consumers defined as those who reported consuming ruminant meat less than once/week at baseline, comparison group n=25, intervention group n=43. *p<.10, **p<.05, ***p<.01.
