# Supplementary material for: Impact of a Scalable, Multi-Campus “Foodprint” Seminar on College Students’ Dietary Intake and Dietary Carbon Footprint
Source: Nutrients. 2020 Sep 22;12(9):2890. doi: 10.3390/nu12092890 (PMC7551495; doi:10.3390/nu12092890)
Supplement: Supplementary file 1 [file nutrients-12-02890-s001.zip › Foodprint S4.docx]

**Appendix I. Open-ended comments (verbatim) from *Foodprint seminar* students, Spring and Fall 2019**

| **Do you intend to do anything differently as a result of this course? Yes (please describe): (n=54)** |
| --- |
| Partly because of this course, I've started having a vegetarian diet. |
| i'm making my family go vegetarian LOL |
| Try to eat less red meat, and to try to make some more things at home like guacamole and hummus instead of buying them |
| Trying to cut out animal products more than I already do, with a focus on reducing cheese consumption |
| I plan on adopting more of a Mediterranean based diet to be more impactful on the environment and human health |
| I intend to eat less packaged foods and eat according to seasonal availability. |
| Consume meat less often and choose better alternatives |
| I’ve been limiting eating meat over the course of the quarter and as of last week I have been eating a vegan diet |
| I intend to share what I learned in this course with my friends and family and try to eat more of a flexitarian diet. |
| I'll be eating less meat and thinking more about my food choices |
| consume less beef |
| At some point in my life, I want to make the transition to becoming a vegetarian. This decision is one that has become more solidified throughout the course as I have learned how large my impact really is |
| I want to focus on limiting my food waste. |
| I will try to go at least one day per week without eating meat products. |
| I have been trying to buy more local produce and also buying less processed foods. |
| Yes, I want to research more about the effects of processed food on health and gut microbiome |
| I want to eat a healthier diet. I consume everything on my plate but want to be more environmentally aware and conservative. |
| Follow a Flexivariant Diet |
| I will be more conscientious with my food choices and try to learn more about where my food comes from before purchasing it. |
| I will focus on having a more plant-centric diet from now on, especially when I'm living on my own and can buy my own food. |
| I intend to eat little to no red meat and go meatless at least twice a week. |
| not eat red meat any more |
| Change my dietary choices to favor reduced meat consumption, and portion my plate to better represent meatless, healthy eating |
| I plan to eat less red meat and substitute it with other animal proteins like chicken or vegetable proteins. |
| Eat less red meat |
| I want to make efforts to contribute to less food waste. |
| Change my eating lifestyle |
| Eat differently, have meatless meals at least twice a week |
| I have told everyone close to me about the things i’ve learned from this course. I have committed to never buying plastic water bottles and I have limited my intake of meat greatly. I also buy the majority of my groceries from a farmers market each week. |
| Change my diet to be more environmentally conscious |
| Make choices in what I am eating to limit my carbon footprint |
| Try to eat even less meat. |
| Be more conscious of my eating choices |
| I intend to be much more conscious of my diet and consumption for the sake of the environment. |
| Choose more local foods |
| Go out and make a difference |
| Limit beef intake to once per week |
| I will try to limit my waste |
| I intend to cut back on my meat intake, especially red meat where the carbon footprint is large |
| I’m going to try to choose vegan options when offered and buy local food |
| I will try to eat less meat and try more vegetarian and vegan options. |
| eat less meat |
| Reduce food waste |
| try to get more people to eat sustainably |
| Limiting more my consumption of vegan alternatives. |
| I intend to no longer eat meat (other than poultry) and to limit my intake of poultry when possible. |
| Consider impacts of even my plant based foods, such as the release of methane from rice. |
| I am going to think more about the larger systems and also consider cheese when trying to reduce my carbon footprint. |
| I plan to really limit my meat intake and spread what I have learned about eating sustainability to my friends and family. |
| Be more aware of the nuances of my eating choices. |
| Eat less meat, including chicken, & in general realize that I don't need to eat as much protein. |
| Avoid beef/pork unless absolutely necessary to consume for cultural purposes. |
| Eat more vegetables and encourage my friends and family to as well. Buy less organic produce. Change my perspective on the danger of GMO foods. |
| Be more conscious of the environmental impact of the food I choose. |

| **The most impactful thing I learned in this course was: (n=57)** |
| --- |
| How much of my environmental footprint is related to my food choices |
| the massive effect one person can have on others/ the environment |
| That the planetary boundaries were a thing. I am glad to know that environmental issues are being measured and we can keep track of how they have changed over the years. |
| The effect that cheese consumption has on the environment |
| The differing impact of agriculture produced in different areas |
| The amount of biodiversity and land loss associated with industrial agriculture systems. |
| How our individual choices we make everyday create big impact |
| Climate change |
| I knew that eating more meat was harmful to the environment, but before taking this class I never really knew the magnitude. I learned the quantitative value of reducing/increasing my meat consumption. |
| The impact of diet on the environment |
| Antibiotics |
| The impact of pesticide resistant DNA found in the soil near ares that grew large amounts of livestock. This just emphasizes the point of how antibiotic resistance is prevalent. It emphasizes the importance of limiting our antibiotic use and the steps we should take to combat the issue |
| Eating less animal products some of the time can add up to a great impact in reducing our environmental impact. |
| Ways to reduce environmental impact |
| I learned how there is a variation in environmental impacts of different categories for different foods, and that meat consumption, which is at a very high rate right now, has been a big factor. People do not necessarily have to switch to a strict vegetarian or vegan diet but can be flexatarian, which still gives a strong impact on reducing the environmental impacts. |
| The most impactful thing that i learned about this course is that change is in my hand and its my responsibility to my health and others to promote environmental friendly choices by my actions and choices. |
| The way we consume and process our foods matter. It's a universal concern |
| The consequence of the meat consumption to climate change |
| About bacteria |
| Our diets have a large environemental impact. Food isn't simply brought straight to dinner tables, there's a long process that comes with it. |
| How almost every aspect of the food industry, whether it be production or consumption is strongly contributing to the negative climate changes we are seeing. However, we have the capability to make changes to prevent this from happening in very realistic ways, which I found to be very powerful and have an incredible influence on how I think about the planet and my choices. |
| We have reached the "red" zone in many places that the average person doesn't even think of. |
| how much of an impact we all have |
| The true impact our consumption and spending habits have on the agricultural impact on the environment |
| Learning about the various footprints created during food production. |
| the impact the food system can have on biodiversity |
| Humans are not likely to make significant behavioral changes unless pushed to the brink |
| How I can make a difference. |
| Footprint |
| Specific environmental foodprints of food categories, specifying what are the critical issues for our Earth (planetary boundaries study) |
| how much convenience contributes to environmental degradation... for example before taking this course I had not even considered that my choice to buy packed, cut-up pineapple had a different environmental footprint than buying a whole pineapple and cutting it up myself at home. the details of refrigeration, transportation, and packaging were not things I thought about before this course. |
| what i should be eating instead of what to avoid eating |
| The amount people can affect climate change by making small dietary changes |
| Rates of biodiversity loss due to food production. |
| Land usage by cattle |
| The comparison of the carbon footprints of third world countries vs. well-developed countries. |
| Factors such as transportation and farming practices also have a big environmental impact |
| How much of a crisis we are currently facing when it comes to the utter destruction of our planet |
| The environmental impact of beef production |
| Impact ruminants have on the environment, even through dairy |
| The importance of what you eat and how it affects the environment |
| The co2 impact based on the food I choose |
| The carbon footprints of various foods and how I could lower my carbon footprint from my food. |
| carbon footprint of meats |
| About antibiotic resistance |
| Land use |
| the power that dietary changes can have on all different boundaries |
| The relationship between almond milk and water usage. |
| The food system is at the core of climate change and presents a nexus of many environmental issues. Reforming the food system would have a significant effect on combating climate change and presents an incredible opportunity to not only make environmental changes, but to fix a global health crisis, as well. |
| That eating a vegetarian diet is only marginally better for the environment than just eating a non-ruminant environment. |
| The nitrogen cycle issue |
| The amount of resources that go into a very small amount of meat. |
| The section about antibiotic resistance was interesting. |
| Understanding that most aspects of climate change can be traced back to issues with raising cattle & red meat |
| The drastic environmental difference between plant and animal protein. |
| The interconnectedness of all of the aspects of the food crisis we are living in and even more so the interdependency of the solutions that are necessary to solve it. |
| The sheer amount of resources consumed and waste produced by beef production. |
